# Whole-Body Diffusion-Weighted Imaging With Background Body Signal Suppression for Primary Tumor Detection in Patients With Brain Metastases

**DOI:** 10.7759/cureus.95971

**Published:** 2025-11-02

**Authors:** Nozomi Hirai, Sho Sato, Morito Hayashi, Shusaku Takahagi, Norihiko Saito

**Affiliations:** 1 Neurosurgery, Toho University Ohashi Medical Center, Tokyo, JPN; 2 Neurosurgery, Ishii Hospital of Neurosurgery ＆ Ophthalmology, Fukushima, JPN

**Keywords:** brain metastasis, case report, diffusion-weighted whole-body imaging with background body signal suppression, esophageal carcinoma, fludeoxyglucose-18 positron emission tomography, lung adenocarcinoma, primary tumor

## Abstract

Diffusion-weighted whole-body imaging with background body signal suppression (DWIBS) is a magnetic resonance imaging (MRI) technique that enables noninvasive, radiation-free whole-body screening. By emphasizing diffusion-restricted regions while suppressing background signals, DWIBS has emerged as a potential alternative or complement to fludeoxyglucose-18 positron emission tomography (FDG-PET), particularly for the detection of primary tumors and metastases. Herein, we report two cases in which DWIBS highlighted suspicious lesions that guided targeted CT and biopsy, leading to the identification of the primary tumor after intracerebral metastasis. The first case involved a man in his 60s who presented with headache and diplopia. Brain MRI revealed clival metastasis, which was diagnosed as squamous cell carcinoma. DWIBS highlighted a focal lesion in the mid-esophagus, which prompted targeted contrast-enhanced CT and subsequent endoscopic biopsy that confirmed esophageal squamous cell carcinoma as the primary site. DWIBS revealed a suspicious lesion in the esophagus, which was later confirmed as an esophageal carcinoma. In the second case, a man in his 50s presented with hemiparesis due to brain metastasis, which was diagnosed as adenocarcinoma. Initial computed tomography and FDG-PET/CT did not localize the primary site; DWIBS subsequently demonstrated a focal diffusion-restricted lesion in the right lung apex, which directed further evaluation and was ultimately consistent with a lung primary. While the initial computed tomography and FDG-PET failed to identify the primary site, DWIBS later detected a lesion in the right lung apex, consistent with lung cancer. These cases illustrate the ability of DWIBS to provide actionable complementary information when FDG-PET is unavailable or yields negative findings. DWIBS, which is accessible, cost-effective, and does not involve radiation exposure, represents a promising imaging modality for oncological practice.

## Introduction

The imaging modality diffusion-weighted whole-body imaging with background body signal suppression (DWIBS) in magnetic resonance imaging (MRI) was first reported by Takahara et al. in 2004 [1], and is based on diffusion-weighted imaging (DWI). Following division of the entire body into multiple segments, diffusion-restricted regions, such as those caused by primary and metastatic tumors, are simultaneously emphasized while suppressing and integrating unwanted background body signals to obtain whole-body images. DWIBS is noninvasive, involves no radiation exposure, and can be performed relatively easily at facilities equipped with MRI systems. It may, therefore, represent an alternative or complement to fludeoxyglucose-18 (FDG) positron emission tomography (FDG-PET) [2,3], which has previously been used for whole-body cancer screening and primary tumor detection, alongside contrast-enhanced computed tomography (CT). FDG-PET, in particular, offers high sensitivity and can detect tumors throughout the body. However, FDG-PET, unlike the morphological imaging modalities CT and MRI, is a functional imaging modality, and the physiological state of the patient thus significantly influences its findings; specific tumor types may not take up FDG. In addition, FDG-PET is expensive, involves radiation exposure, and is less widely performed than CT or MRI, with its use varying according to region and facility. There is, therefore, a growing need for a more convenient and noninvasive whole-body imaging modality. Nevertheless, FDG-PET is not uniformly reliable across all clinical scenarios. Indolent or well-differentiated malignancies, certain low-uptake lung adenocarcinomas, and very small apical lung lesions may demonstrate little or no FDG accumulation because of limited glycolytic activity, partial volume effects, or respiratory motion. As a result, FDG-PET can yield false-negative findings even in patients who already present with metastatic disease, including brain metastasis [4]. In such settings, there is an urgent need for an alternative whole-body survey that can localize suspicious lesions without relying solely on glucose metabolism and that can rapidly support initial staging and treatment planning.

This study reports two patients in whom DWIBS provided clinically useful leads by localizing suspicious lesions that then guided targeted CT and biopsy to identify the primary tumor. This report focuses on cases where symptoms first manifested due to metastatic brain lesions, even when the primary tumor was asymptomatic, necessitating immediate identification of the primary site. MRI is relatively accessible at many medical institutions, and DWIBS may be feasible even at facilities or in regions where FDG-PET is not readily available. This report aims to detail the clinical context in which DWIBS was performed, and its contribution to decision making, rather than to claim diagnostic superiority over other imaging modalities. We also summarize the technical parameters used for DWIBS to facilitate reproducibility and discuss its limitations relevant to clinical practice.

## Case presentation

Imaging protocol

DWIBS was performed on a 1.5T SIGNA Explorer MRI system (GE HealthCare, Chicago, IL, USA) using single-shot echo-planar imaging and short T1 inversion recovery fat suppression without respiratory gating. The parameters used were as follows: b-value, 800 s/mm²; repetition time, 6,081 ms; echo time, 84.8 ms; inversion time, 180 ms; matrix, 108×128; field of view, 50×35 cm; slice thickness, 6 mm. Images were acquired at four stations, for 3 min 35 s per station, repeated once; T1-weighted images were acquired to support anatomical fusion. In the post-processing stage, maximum intensity projection (MIP) reconstruction was performed on the vendor-supplied workstation. No custom or research-only software was required; the MIP images were generated using the standard scanner software. The apparent diffusion coefficient was not measured. For artifact handling, parallel imaging and a wider bandwidth were used to mitigate susceptibility and motion, acknowledging the reduced performance near the lungs and heart. This protocol can be implemented on a standard clinical 1.5T scanner with multi-station DWI capability and Short Tau Inversion Recovery (STIR) fat suppression; no specialized hardware beyond routine whole-body DWI and standard MIP reconstruction software is required.

Case one

**Figure 1:**
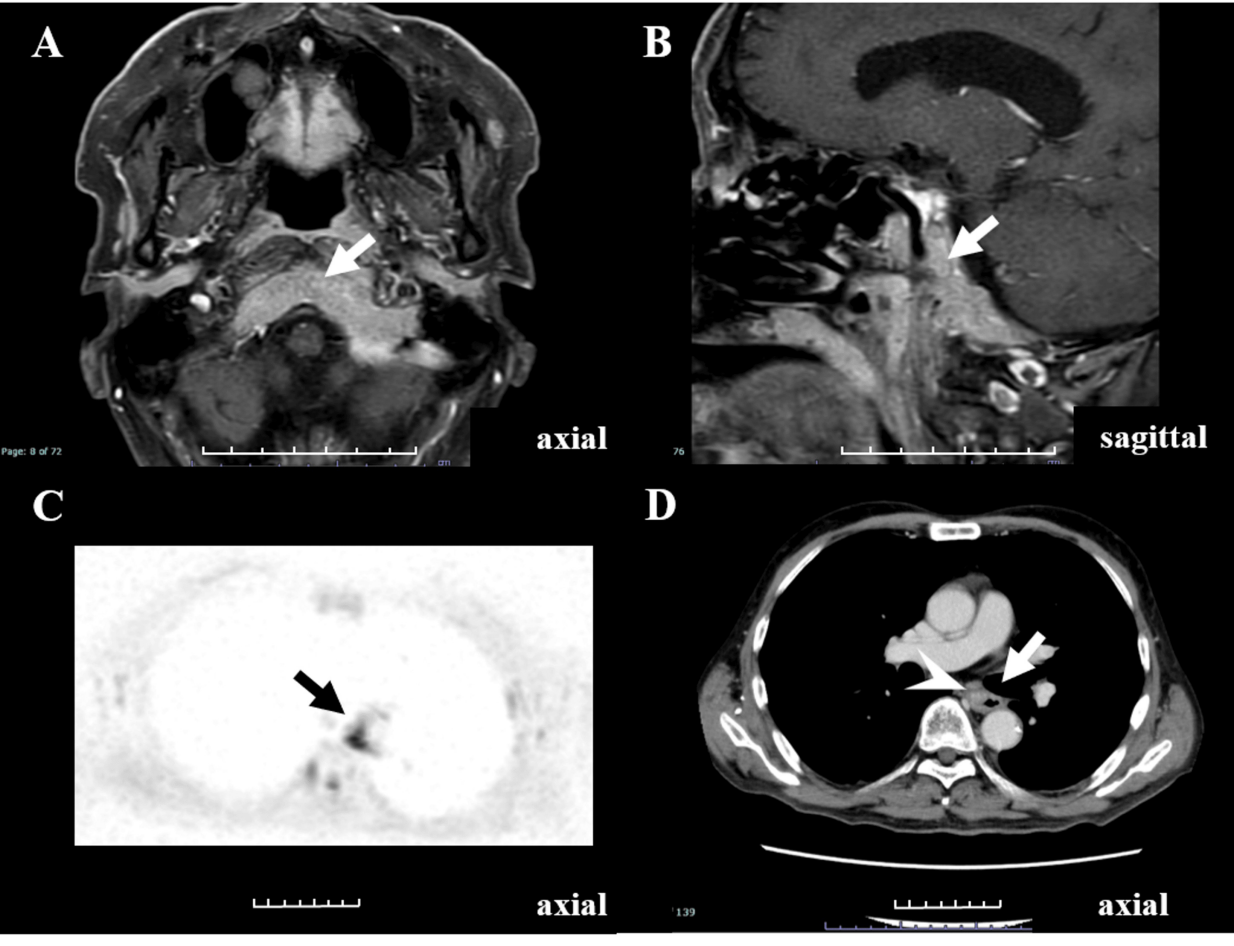
Brain MRI, DWIBS, chest contrast-enhanced CT images Initial (A) axial and (B) sagittal contrast-enhanced T1-weighted MRI. The clivus shows uniform enhancement, suggesting metastasis (white arrow); (C) Diffusion-weighted whole-body imaging with background body signal suppression (DWIBS) with b=800 s/mm² reveals high signal intensity (black on this image) in the esophagus (black arrow); contrast-enhanced chest computed tomography shows contrast enhancement in the same region, suggesting esophagus cancer (primary tumor); (D) White arrowhead indicates enhancing esophageal wall thickening with suspected adventitial extension. The lesion is indicated in each panel by an arrow. Each division on the scale bar is 1 cm.

A man in his 60s presented with headaches and diplopia. Brain MRI revealed a contrast-enhancing lesion in the clivus (Figures 1A, 1B).

Transnasal endoscopic biopsy of the clival lesion demonstrated metastatic squamous cell carcinoma. Because the patient presented with intracranial metastasis and the primary site was unknown, whole-body DWIBS was obtained early as part of our institution’s initial workup for brain metastasis of unknown origin. Whole-body DWIBS highlighted a focal lesion in the mid-esophagus, which prompted targeted contrast-enhanced CT and subsequent endoscopic biopsy that confirmed esophageal squamous cell carcinoma as the primary site (Figure 1C). Targeted contrast-enhanced chest CT showed a corresponding approximately 20 mm, contrast-enhancing wall-thickening lesion in the mid-esophagus, predominantly along the right and anterior walls (Figure 1D). Based on these concordant imaging findings, an endoscopic biopsy of the esophageal lesion was performed and confirmed squamous cell carcinoma of the esophagus. The final diagnosis was esophageal squamous cell carcinoma (Mt), cT4N2M1, stage IVB. Palliative radiation therapy was initiated for symptom control, including relief of cranial base pain and diplopia related to the clival metastasis, in the setting of stage IV disease, while systemic therapy was considered the standard of care. A summary of case one is presented in Table 1.

**Table 1:** Summary of the cases DWI: diffusion-weighted imaging; DWIBS: Diffusion-weighted whole-body imaging with background body signal suppression; FDG-PET: fludeoxyglucose-18 positron emission tomography; MRI: magnetic resonance imaging; CT: computed tomography.

Summary of the cases	Case 1	Case 2
Patient characteristics		
Age/Sex	60s / male	50s / male
Presenting neurological symptoms	headaches and double vision	paralysis of the left side of the face and the left upper and lower limbs
Pathology	metastatic squamous cell carcinoma	metastatic adenocarcinoma
Key imaging findings across modalities		
Brain MRI	The clivus shows uniform enhancement, suggesting metastasis.	A 2.5 cm contrast-enhancing lesion is noted in the right middle frontal gyrus, suggesting metastasis.
Contrast-enhanced CT of the chest and abdomen	Contrast-enhanced chest CT shows contrast enhancement in the esophagus, suggesting esophagus cancer.	Initial chest CT reveals emphysematous changes in the right lung apex. Four months later, a chest CT scan revealed enlargement of the lesion in the right lung apex, suggesting lung cancer (primary tumor).
FDG-PET	N/A	FDG-PET CT performed concurrently with the initial chest CT shows no uptake of fludeoxyglucose-18 in the right lung apex.
DWIBS	DWI reveals high signal intensity (black on this image) in the esophagus	DWIBS performed four months after the initial chest CT revealed high signal intensity (black on this image) .
Timeline		
Onset of neurological symptoms	Day 0	Day 0
First contrast enhanced brain MRI	Day 1	Day 1
Pathology confirmation of brain metastasis	Day 60	Day 16
Systemic imaging (DWIBS / chest CT/ FDG-PET)	DWIBS: Day 62 / chest CT: Day 63 / FDG-PET：N/A	DWIBS: Day 120/ chest CT: Day 21 /FDG-PET: Day 51
Identification of presumed primary	63 days	120 days

Case two

**Figure 2:**
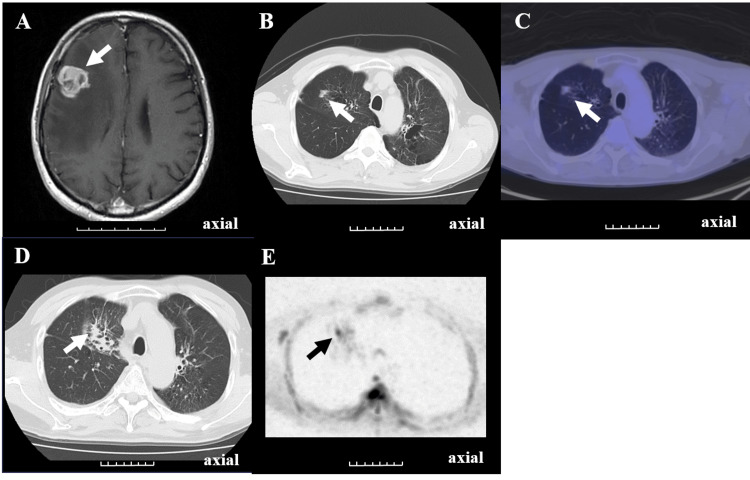
Brain MRI, chest CT, PET-CT, DWIBS images (A) Contrast-enhanced T1-weighted magnetic resonance imaging (MRI) reveals a 2.5cm ring-shaped contrast-enhanced lesion in the right frontal lobe, suggesting brain metastasis (white arrow); (B) Initial chest CT reveals emphysematous changes in the right lung apex (white arrow); (C) while fludeoxyglucose-18 positron emission tomography (FDG-PET) shows no uptake of fludeoxyglucose-18 (SUVmax: 1.3) in the same area (white arrow); (D) Chest computed tomography (CT) performed four months after initial chest CT demonstrates enlargement of the lesion in the right lung apex, suggesting lung cancer (primary tumor) (white arrow); (E) Diffusion-weighted whole-body imaging with background body signal suppression (DWIBS) with b=800 s/mm² performed four months after the initial chest CT shows high signal intensity (black on this image) in the same area (black arrow). The lesion is indicated in each panel by an arrow. Each division on the scale bar is 1 cm.

A man in his 50s presented with paralysis of the left side of the face and the left upper and lower limbs. Head MRI revealed a 2.5 cm contrast-enhancing lesion in the right middle frontal gyrus (Figure 2A).

Due to the paralysis experienced by the patient and the significant peritumoral cerebral edema observed, tumor resection was performed, resulting in a pathological diagnosis of metastatic adenocarcinoma. A whole-body CT scan performed to determine the primary cancer site revealed emphysematous changes in the right lung apex (Figure 2B). Initial FDG-PET (non-respiratory-gated) showed no FDG uptake (SUV max: 1.3) in the right lung apex (Figure 2C), possibly due to the small size of the lesion and respiratory/partial-volume limitations. Four months later, a new metastatic brain tumor was identified and chest CT revealed enlargement at the same site (Figure 2D). Because the primary site still remained undetermined despite CT and FDG-PET/CT, whole-body DWIBS was then performed as an adjunct problem-solving study to further evaluate for an extracranial primary lesion. DWIBS showed a concordant focally high signal (Figure 2E). Following targeted follow-up, the patient was diagnosed with primary lung adenocarcinoma. A summary of case 2 is presented in Table 1.

## Discussion

In the cases described in the present study, DWIBS helped identify the primary esophageal and lung tumors that were discovered following intracranial metastases. Metastatic brain tumors occur in 10-20% of patients with malignant tumors and are often a critical prognostic factor [5-7]. Lung cancer is the most common primary cancer in cases of brain metastasis, followed by breast, gastrointestinal, and renal cancers. Historically, median overall survival after the diagnosis of brain metastasis was on the order of only a few months [6]; however, outcomes have improved in selected patients due to advances in systemic therapy (including targeted and immune-based regimens), stereotactic radiosurgery, and multidisciplinary management.

Prompt identification of the primary tumor is clinically essential to direct the treatment strategy. Recent studies have reported that primary tumors and distant metastases exhibiting diffusion restriction can be identified using DWIBS [8]. This method is applicable not only to cancers with markedly restricted diffusion, such as lymphomas and small cell tumors, but also to many common cancer types [2,8,9]. DWIBS is currently used for cancer diagnosis and staging, as well as for evaluating the treatment response in patients with prostate and breast cancers [10]. However, motion artifacts make it difficult to evaluate areas near cardiac or diaphragmatic regions using DWIBS.

In this case of esophageal squamous cell carcinoma, DWIBS showed a focal high signal, and the corresponding CT suggested transmural involvement, including probable adventitial extension. Conversely, CT often only reveals changes in wall thickness, which may make lesion identification difficult. Although FDG-PET indicates the extent of FDG uptake, the borders are often indistinct, and DWIBS has been reported to be superior in accurately determining the anatomical extent of a tumor. DWIBS detection rates of esophageal cancer according to depth of invasion have been reported as follows: T1a, 0%; T1b, 42.9%; T2, 50%; T3, 100%; and T4, 100%. Corresponding FDG-PET detection rates have been reported as: T1, 43%; T2, 90%; T3, 98%; and T4, 100% [11,12]. Although both methods have limitations in detecting early-stage lesions, the detection rates for advanced cases are high. The case of esophageal cancer described in this report was T4, explaining its detection by DWIBS. In this patient, DWIBS functioned as an initial whole-body screen that focused subsequent imaging and biopsy. 

In selected contexts, whole-body DWI and DWIBS have shown comparable performance to FDG-PET for detecting primary lesions and evaluating lymph nodes in patients with NSCLC; however, results vary according to tumor type, lesion size, and acquisition quality [13,14]. DWIBS is helpful in facilities where FDG-PET is unavailable, or in cases where no FDG uptake is observed. In the second case described in this report, the apical lung lesion showed no uptake on FDG-PET, but was detected using DWIBS. This suggests that DWIBS may be useful in cases of false-negative FDG-PET results. FDG-PET false negatives may occur with small/apical lung lesions because of respiratory blurring and partial volume; tumor biology and glycemic status may also influence FDG uptake. The false negative rate for lung cancer on FDG-PET is generally reported to be 8-15% [15]. This underscores the role of DWIBS as a complement to, rather than a replacement for, FDG-PET. In small apical lung lesions (<1 cm), FDG-PET may yield false negatives due to respiratory motion and partial volume effects, and DWIBS can also be limited by susceptibility and motion in the thorax. These modalities should therefore be viewed as complementary rather than mutually exclusive.

The advantages of DWIBS include its non-invasive nature and lack of radiation exposure, its ability to achieve whole-body scanning without contrast agents, its wide applicability owing to its compatibility with all MRI systems, and its lower cost compared to FDG-PET. However, challenges remain, including the complexity of evaluating organs containing significant air, such as the lungs, or organs with movement, such as the heart; the high background signal intensity of organs such as the spleen and testes; and insufficient standardization in image interpretation, which leads to variations in findings between facilities. In recent years, the usefulness of DWIBS for detecting bone metastases in patients with prostate cancer has been recognized by guidelines issued in Japan, and its applications are expanding [16]. Further improvements in diagnostic accuracy are expected to result from the combination of FDG-PET and DWIBS. Organs showing physiological accumulation on FDG-PET do not necessarily correspond to those showing physiological diffusion restriction on DWIBS. As FDG-PET and DWIBS reflect distinct phenomena, FDG-PET/MRI may compensate for the disadvantages of each modality.

This study has several limitations. First, it is a small, retrospective case series from a single institution, so the findings are descriptive and may not be generalizable. Second, imaging was performed on different scanners with non-uniform acquisition parameters (MRI, DWIBS, CT, and FDG-PET), which limits direct quantitative comparison across cases and modalities. Third, quantitative metrics such as lesion size, apparent diffusion coefficient (ADC), and FDG-PET SUVmax were taken from contemporaneous clinical radiology reports only when they were explicitly documented. In several lesions these values were not recorded as part of routine clinical interpretation, and are therefore listed as “not available” in the tables. We did not retrospectively re-measure ADC or SUVmax, because post hoc measurements across different scanners and reconstruction methods would not be technically standardized and could be misleading. Finally, the diagnostic timelines we report (intervals between neurological presentation, brain imaging, histologic confirmation, and systemic imaging) reflect real-world clinical decision-making rather than a predefined workup protocol. Thus, our cases illustrate a pragmatic pathway by which brain metastasis can reveal an otherwise occult primary tumor, but they should not be interpreted as prescriptive diagnostic guidelines.

## Conclusions

In the two patients with brain metastases described in this report, DWIBS yielded information that facilitated primary tumor identification: one esophageal lesion and one apical lung lesion that was not detected by FDG-PET. DWIBS should be considered as an adjunct when FDG-PET results are negative or FDG-PET is unavailable. Claims of superiority are beyond the scope of case reports and require controlled comparative studies.

These findings suggest that DWIBS may offer additional diagnostic value in selected cases, particularly when conventional imaging provides limited information.
